# Improving cardiac rehabilitation patient adherence via personalized interventions

**DOI:** 10.1371/journal.pone.0273815

**Published:** 2022-08-29

**Authors:** Keren B. Aharon, Avital Gershfeld-Litvin, On Amir, Irene Nabutovsky, Robert Klempfner

**Affiliations:** 1 Co-Founder and Chief Scientific Officer, Well-Beat, Kfar Saba, Israel; 2 The Academic College of Tel Aviv-Yaffo, Yaffo, Israel; 3 UC San Diego, Rady School of Management, La Jolla, San Diego, CA, United States of America; 4 Sheba Medical Center, Ramat Gan, Israel; 5 Sheba Innovation Center, Ramat Gan, Israel; University of Florida, UNITED STATES

## Abstract

**Objectives:**

Despite documented benefits and physicians’ recommendations to participate in cardiac rehabilitation (CR) programs, the average dropout rate remains between 12–56%. This study’s goal was to demonstrate that using personalized interventions can significantly increase patient adherence.

**Method:**

Ninety-five patients (ages 18–90) eligible for the CR program were randomly recruited and received personalized interventions using the Well-Beat system. Adherence levels were compared to those of a historical control group. The Well-Beat system provided Sheba CR Health Care Provider (HCP) guidelines for personalized patient-therapist dialogue. The system also generated ongoing personalized text messages for each patient sent twice a week and related each patient’s dynamic profile to their daily behavior, creating continuity, and reinforcing the desired behavior.

**Results:**

A significant increase in patient adherence to the CR program: Three months after initiation, 76% remained active compared to the historical average of 24% in the matched control group (log-rank p-value = 0.001).

**Conclusions:**

Using an Artificial Intelligence (AI)-based engine that generated recommendations and messages made it possible to improve patient adherence without increasing HCP load, benefiting all. Presenting customized patient insights to the HCP and generating personalized communications along with action motivating text messages can also be useful for remote care.

## Introduction

Cardiovascular disease (CVD) is a leading cause of death worldwide (AHAACC). The burden of CVD also reflects in life-years lost, diminished quality of life, and direct and indirect medical costs [1]. Previous studies have shown that cardiac rehabilitation (CR) improves survival, cardiovascular morbidity and mortality, re-hospitalization, exercise capacity, lifestyle habits, and psychosocial well-being [2]. CR is a multi-component intervention program provided by multidisciplinary HCPs. The program includes monitored physical activity, medical supervision, patient education, and if needed, consultations with a psychologist and a nutritionist. This program is an important component in the prevention of complications from heart disease [3]. Studies have shown that only about half of the countries in the world offer CR programs [4].

Despite documented benefits of CR, participation rates in CR programs in the United States are only 20% to 30% [5]. Of the patients starting the rehabilitation program, 24–50% drop out and are therefore unable to achieve the desired outcome [6, 7]. Lack of participation is caused objective barriers, such as distance, lack of accessible transportation, travel cost and uncomfortable hours of work, associated costs, and lack of sufficient insurance coverage, as well as well as behavioral and psychosocial reasons [8, 9].

In light of this, the study’s primary aim was to test whether using personalized interventions can significantly increase patient adherence (measured at 3- and 6-months post CR initiation) compared to a matched historical control group. Secondary aims included evaluation of feasibility, in terms of Health Care Providers (HCP) satisfaction and system usability.

## Method

### Participants

#### Patients

The study included patients discharged from a cardiovascular event or procedure related hospitalization who were eligible for CR at the Sheba CR center, a unit located in the Sheba Hospital. This unit includes a gym, examination rooms, and treatment rooms for the rehabilitated. The study focused on gym activity attendance. A total of 106 patients chosen at random were asked to join the study and signed a consent form. Ten were disqualified due to non-compliance with the threshold conditions and another participant left the program due to medical reasons shortly after being recruited. Thus, the final sample included 95 patients. Study enrollment was performed during the on-site pre-rehabilitation lecture as well as the first month of activity in the CR program (see Table 1 for demographics). All patients were entitled to cardiac rehabilitation by Health Maintenance Organization (HMO) policy (the paying entity) and lived up to an hour’s drive from the center. For 66% of the patients (70 patients), this was the first cardiovascular hospitalization. The CR Center enrollment comprises several hundred patients at any given point in time. A small percentage are center veterans (one or more years) and the majority have been enrolled for less than one year. Several dozen new patients join monthly, while others leave. At any point in time during the study, the study participants accounted for less than a third of the patients who trained at the center.

**Table 1:** Patient demographics and prior diagnoses.

Variable	Test group	Control group	Difference
	N = 95	N = 500	
Age^a^	61.31	65.41	*P* = .07, ns.
Gender	21.3%^b^	23.9%^c^	*P* = .65, ns.
Type 2 Diabetes Mellitus	32.9%	24.6%	*P* = .09, ns.
Hypertension	59.5%	54.3%	*P* = .35, ns.
Past Cerebrovascular Accident (CVA)	10.6%	5.21%	*P* = .04
Past Transient Ischemic Attack (TIA)	7.45%	4.00%	*P* = .14, ns.
Heart Failure	18.1%	12.6%	*P* = .16, ns.
Myocardial Infarction (MI)	40.4%	36.2%	*P* = .44, ns.
Coronary Bypass Surgery (CABG)	28.7%	24.2%	*P* = .36, ns.
Atrial Fibrillation	21.2%	16.0%	*P* = .21, ns.
Past Valve Replacement	13.8%	14.2%	*P* = .92, ns.

^a^Standard deviation for Age was 14.6 in the test group and 10.4 in the control group.

^b^Percentage of female subjects in the test group.

^c^Percentage of female subjects in the control group.

A historical comparison group was generated from data on 500 patients, randomly chosen from the parallel period in previous years (2017–2018), using a propensity score matching procedure that included, among other variables, age, gender, and rehabilitation indicators (see Table 1). The adherence of the study participants was compared to this historical control group.

#### HCPs

The team included 22 HCPs in a variety of specializations: physicians, nurses, fitness trainers, exercise physiologists, and bio-technicians (see Table 2). Over 90% of them (20 HCPs) had years of experience as experts in their respective fields. All gym HCPs participated in the study. In addition, two physicians were recruited, although not included in the gym staff. Those physicians usually see the patients at the beginning and end of the rehabilitation process. The in-depth interviews at the end of the study included HCPs who happened to be present at the center on the day of the interviews which was determined randomly.

**Table 2:** HCP participants.

HCP specialization	N	Percentage
		(Out of HCP)
Fitness trainers	7	0.27
Exercise physiologists	3	0.32
Bio-technicians	4	0.18
Nurses	6	0.14
Physicians	2	0.09
	22	

### Procedure and design

#### Participants recruitment

We posted an ad inviting participation in the study at the lecture hall for a new patient briefing. The Ad read (translated from Hebrew): "New VIP service. We create custom communication for you. To join the study, contact the cardiac rehabilitation team." Recruitment was done first come first served basis: The first 106 patients who met the screening criteria were admitted after signing a consent form. Screened participants needed to be smartphone holders entitled to rehabilitation at no additional charge for at least two months from the date of joining the study.

#### Admission of participants

Adding patients to the study was performed in two steps. First, patients were presented with the purpose of the study and the six months duration. The research coordinators offered them to participate voluntarily, and consenting patients signed an informed consent form. Second, each participant was given assigned an anonymized IDs, through which they were identified in the system, to maintain their privacy.

#### Initial information gathering

After joining the study, a digital questionnaire assessing their initial state was sent to each participant via a text message that included a link. This questionnaire was developed by Well-Beat behavioral science experts. If the patient agreed to participate in the study but did not complete the questionnaire, reminders were sent to encourage them to do so (a maximum of five messages were sent).

#### Patient diagnosis

**Fig 1:**
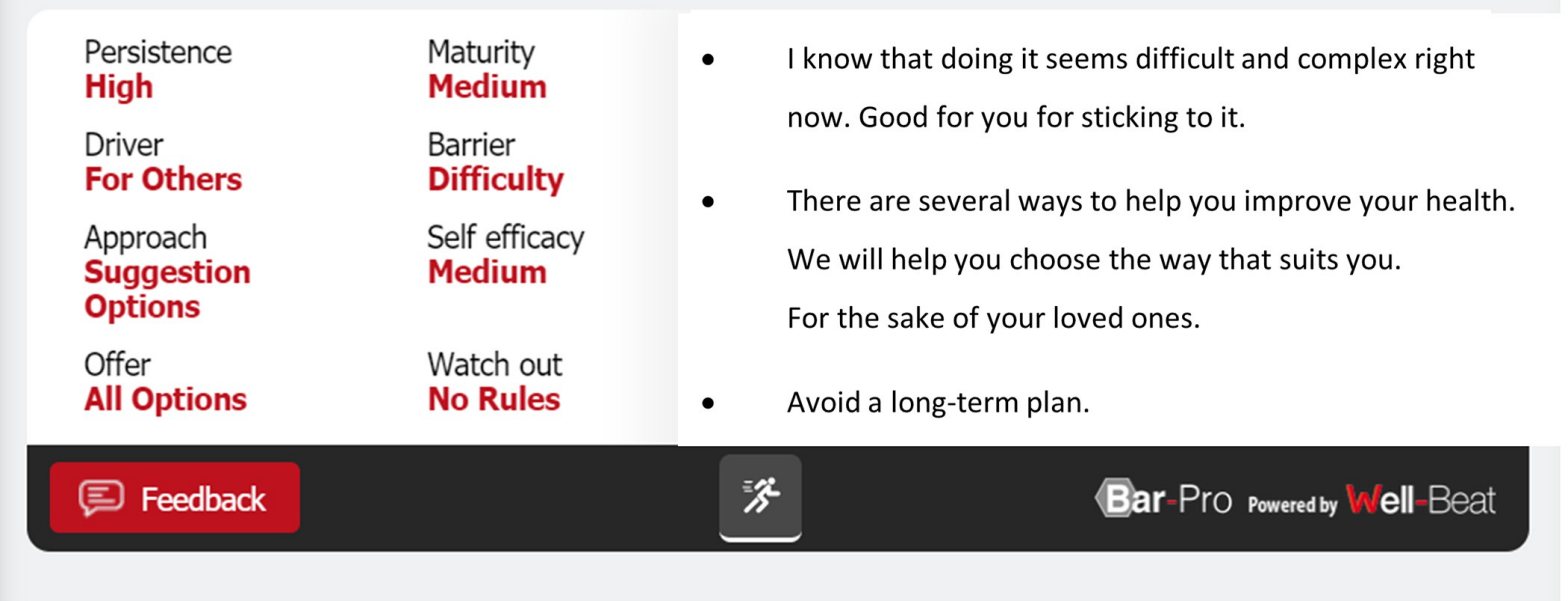
Example of the HCP toolbar for a particular patient.

After processing the questionnaires, Well-Beat’s engine created a profile for each patient and presented it on the HCPs’ Toolbar (see Fig 1). The Toolbar included the patient’s persistence level, readiness for change level (maturity), self-efficacy level, main motivational driver, and barrier, as well as what the HCP should watch out for in communication with them. Based on this information, the engine recommends the personalized patient dialogue to use, in terms of the tone, the style, and motivation factors, as well as what to avoid saying. There was no reference to the medical protocol of the rehabilitation program, as the intervention focused only on the behavioral and communicative aspects. No additional interventions took place with patients who participated in the study. The HCPs accessed the Well-Beat Toolbar using their mobile devices or desktop computers, allowing broad accessibility and availability throughout the office and exercise areas.

#### HCPs use of the system

The HCPs looked at the Toolbar, saw the system recommendations for each patient, and navigated their conversations accordingly while encouraging them to exercise. During the first two weeks, a Well-Beat representative accompanied the HCPs and provided "on-the-job training". The HCPs were asked to fill out a short feedback form after each use, enabling us to verify that they indeed used the Toolbar. The HCPs reported following the Toolbar recommendations. At the end of each HCP-patient interaction, HCPs provided feedback as to the accuracy of the patient profile as displayed in the Toolbar, enabling dynamic accuracy improvements. In cases of conflicts between the feedback provided and the system profile, a partial update was made: Well-Beat referred to it as "expert in the middle" and weighted it into the recommendation engine so that it is taken into account in the daily profile calculation. (Daily updates incorporate patient daily behavior into the recommendation model). For reasons of confidentiality and NDA we cannot discuss details of the updating algorithm itself.

#### Well-Beat’s personalized interventions

The system development was based on previous literature and dozens of interviews with therapists in various fields, including clinical and medical psychologists. Model validation involved 9 qualitative and quantitative field studies that included thousands of patients. Unfortunately, for reasons of confidentiality and NDA, it is not possible to reveal the model and the algorithm.

The Well-Beat system provides a toolbar for the HCPs, displaying guidelines for personalized patient dialogue. Informed by Well-Beat’s recommendation engine, the toolbar relates each patient’s dynamic profile to their daily behavior. Given the importance of creating habits and fostering intrinsic motivation, the system generates ongoing personalized text messages for each patient that are sent twice a week, creating continuity and reinforcing desired behaviors.

#### Alerts

If a patient was absent 3 times in a row, another algorithm in Well-Beat’s engine raised a ’flag’ for a personalized conversation based on the engine’s recommended call script. In the first month, such calls were made by a nurse. From the second month and onwards the calls were made by a fitness trainer.

**Personalized motivational digital messages sent to each patient.** Additionally, twice a week, each patient received personalized motivational digital messages on their smartphone. The engine chose the specific message according to the patient’s profile and the CR attendance record.

#### Satisfaction

At the end of the study, anonymous satisfaction surveys were sent to the HCPs. Moreover, five in-depth personal interviews were conducted with the HCPs and the operations manager to obtain qualitative and detailed feedback on the use of the Toolbar. The HCPs interviewed were selected by the director of the CR center to represent different professional roles (i.e., nurse, fitness trainer, bio-technician, medical physiologist, and director). The qualitative in-depth included three questions:

1. What do you think about using the Toolbar?
2. What would you recommend changing or improving?
3. Please provide examples of the manner in which you used the Toolbar.

In addition, ten in-depth personal interviews were conducted with all patients that were present on a specific date. They were asked about their opinion regarding the effect of the text messages (both positive and negative effects) and the extent to which the messages were suitable for them.

### Patient adherence measurement

In the context of the Sheba Heart Rehabilitation Center, adhering means attending the cardiac rehabilitation center activity twice a week consistently. To enable this, the patient has to pay for the coming month or provide proof of coverage from the insurance company. Patient adherence for both the study and the control group was measured by the CR center’s keycard-activated electronic gate, as well as by insurance reimbursement forms submitted. Note, the first time each month the patient enters the center, he or she activates the reimbursement form regardless of the number of times they participated that month. This implies that tracking the entrances via the electronic gate is more conservative measure of adherence than tracking reimbursement forms. With that being said, the results below refer to reimbursement forms, as the data for this measure is more reliable and complete.

We compared the adherence of the 95 study participants to 500 patients in parallel periods in previous years (2017–2018), creating a Propensity Matched Population with respect to age, gender, and rehabilitation indication. The reason for choosing a historical control group is the inability to isolate the impact of using the insights and the communication tools on research participants alone. To avoid the bias that arises from influencing the HCP behavior and sensitivity, a historical control group was chosen.

Importantly, in Israel, the first three months of CR are fully paid for by the insurer (HMO), and in the following months, the patient is responsible for a small copay. Therefore, three months is defined as the period of patient adherence and there is a predictable (and understandable) drop after three months. Moreover, Well-Beat added the measure of the number of monthly visits to the center, which has not been examined in previous years.

### Ethical considerations

Well-Beat is Health Insurance Portability and Accountability Act (HIPAA) and General Data Protection Regulation (GDPR) compliant. All patients were identified using a random code issued by the cardiac rehabilitation center and the study was approved by the Institutional Review Board at Sheba Medical Center (SMC-5556-18) with consent obtained from all participants.

## Results

### Patient adherence

#### Adherence to the CR program

**Fig 2:**
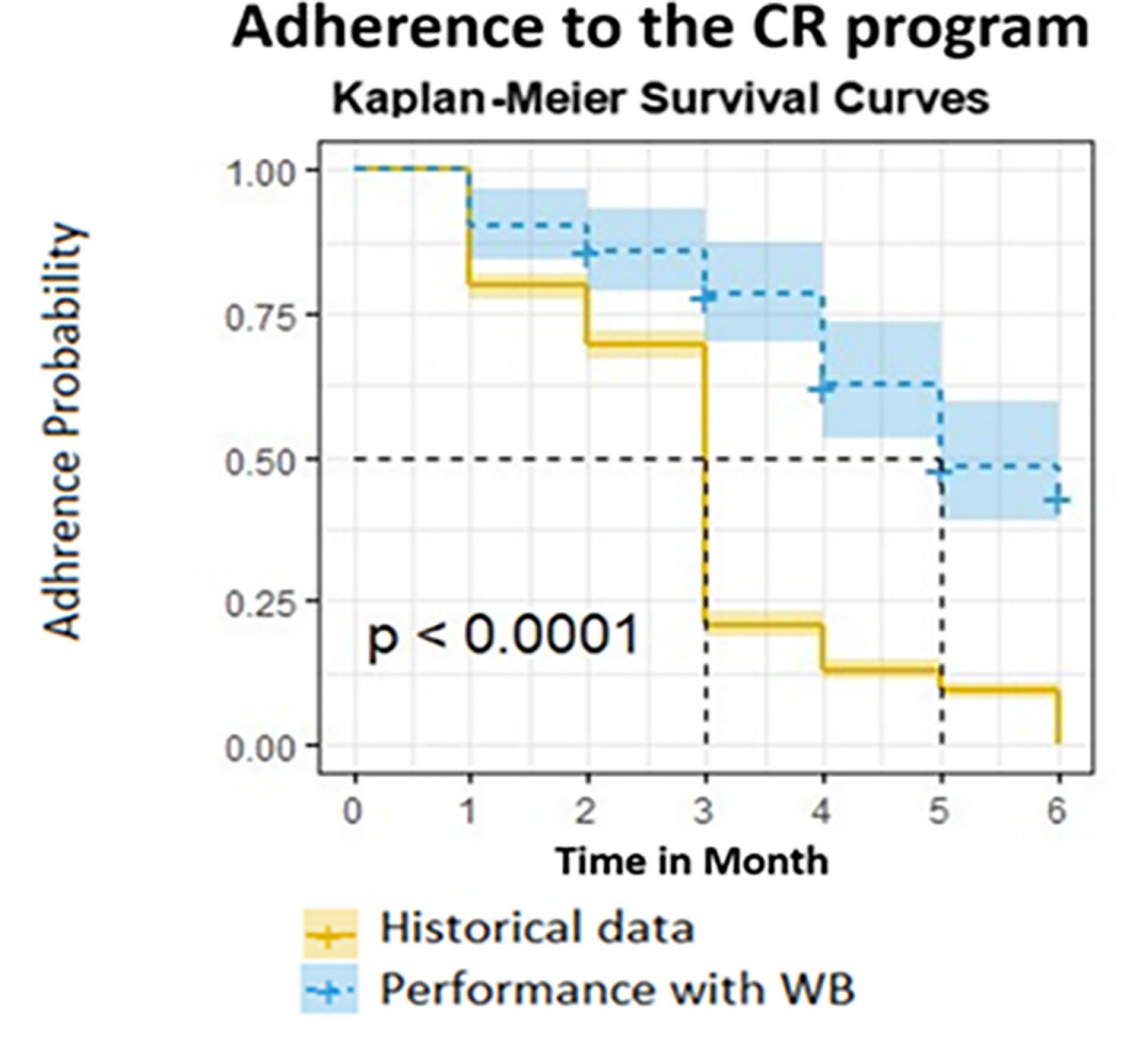
Adherence to the CR program.

As mentioned above, adherence is measured by the monthly submission of insurance reimbursement forms. The use of the Well-Beat system led to a significant increase in patient adherence to the CR program, compared to the control group, as presented by Kaplan-Meier curves in Fig 2. Patients in the Well-Beat program were significantly more likely to continue participating in the CR program at 3 months (*76%*) compared to the historical control group (*24%*, *log-rank p <*0.001). This pattern of increased adherence continued throughout the 6 months of the study, with an overall increase of about 300%.

### Additional findings

#### Initial patient first engagement

Overall, 94% of patients who signed a consent form to participate in the study, completed the initial questionnaire. Eighty-seven percent of participants did so in response to three text messages (see Appendix A for further details). The exit in-depth interviews revealed that those patients aware of the difficulty in perseverance and those who felt the need ‘to be seen’ were more likely to respond.

#### Patient perceptions

An analysis of the initial questionnaire revealed that only 51% of patients participating in the CR program perceived the in-person sessions as necessary (53 patients), while the rest considered in-person appointments desirable but not necessary (40% - 42 patients), as valuable as remote appointments (7% - 8 patients), or undesirable (3% - 3 patients). These results support the promotion and development of remote medical services. It should be noted that the study was done before the COVID-19 pandemic which accelerated the use and acceptance of remote medicine.

#### HCP perceptions

Most of the HCPs that took part in the project (97% - 21 HCPs), including nurses, trainers, medical technicians, medical physiologists, and physicians, perceived the Toolbar as beneficial, and 75% of them found the tool "very helpful" (17 HCPs). Moreover, the percentage of recommendations to implement the Toolbar for daily use was extremely high (see Appendix C for further details). These results are especially impressive given the early skepticism expressed by some of the staff with respect to the Toolbar itself, thinking their experience and intuition would be more accurate.

After each intervention, HCPs who had a face-to-face dialog with a patient provided feedback about the accuracy of the recommendation presented in the Toolbar. Within 14 weeks of activity, 440 feedback entries were received. The HCPs perceived the approach, self-efficacy, maturity, and main driver barrier to be accurate over 90% of the time (93–99% for various parameters) and the motivational barrier parameter accuracy to be around 86%.

## Discussion

Most previous studies associated depreciating patient participation from CR programs with clinical factors and logistical factors (the need to return to work, the financial burden, inconvenient times, transportation issues, and lack of physician referrals), intrapersonal factors (socioeconomic, self-efficacy, and ethnic differences), interpersonal factors (marital and employment status), denial of illness severity, and the CR program itself [5, 8, 9]. Other studies highlight financial constraints or lack of reimbursement by the health insurer [2]. However, in Israel, all citizens have health insurance mandated by law, and in most relevant cases, CR is free of charge for the first three months. Following this period (usually about 26 sessions), most patients (about 80%) can continue CR for an additional nine-month period during which they are afforded a partially subsidized cost [2]. This makes the context of the reported study cleaner to test the effect of behavioral interventions, as there are fewer sources of noise.

The Well-Beat platform is partially based on a well-reviewed model of psychological stress appraisal and coping that was developed by psychologists [10]. Stress arises when individuals perceive that they cannot cope with the demands being made on them or with threats to their well-being [11]. Many medical conditions can be exacerbated by the stress (migraines, ulcers, coronary heart disease, asthma, arthritis, Crohn’s, cancer, and more) and some can even be caused by its enduring effects on the body [12]. However, stress is multi-faceted and not entirely negative. Yerkes and Dodson (1908) found that medium levels of stress can, in the form of arousal, improve performance as compared to low or high levels of stress [13]. The Well-Beat platform harnesses this knowledge and applies it to help patients remain active and committed to the process, despite the stress they may be experiencing and even taking advantage of it.

According to Lazarus and Folkman’s model (1984), when a person encounters a stressful situation (like being diagnosed with an illness), they go through two levels of stress appraisal to assess their situation. First, they think about what it all means; a stage is better known as primary appraisal [10]. This is where a person figures out whether they are facing harm or loss, a threat, a challenge, or other. Later, in the secondary appraisal, they figure out whether they feel that they can cope with the situation, which in turn determines, to some extent, how they cope with it. The theoretical model of stress appraisal and coping is implemented in the Well-Beat platform as a state-space system, where each state represents different levels of the key variables calculated. These states (or patterns) are based on a unique combination of elements developed exclusively by a diverse team of experts who represent a variety of relevant fields including cardiology, endocrinology, nutrition, and psychology. Efforts were made to ensure that appropriate input was given by the experts to contribute to the validity of the platform. The actual implementation details are proprietary and cannot be published.

The use of web-based and mobile applications, telephonic coaching, hand-held computer technologies, the internet, and various wearable activity-tracking devices (e.g., pedometers and accelerometers) provides opportunities to regularly engage CR patients with Health-Lifestyle messages and interventions, an approach that has the potential to substantially increase accessibility, reduce costs, and improve outcomes [14]. To influence patient adherence to CR programs, different strategies have been tested. Research on patient adherence divides interventions into several categories–psycho-education, cognitive-behavioral, regime management, and patient adherence promoting incentives.

There is evidence that psychoeducation as ’Learning and Coping Strategies’ (LC) improved patient adherence to CR [15]. Patients enrolled in CR who participated in a text-messaging program attended more sessions and were significantly more likely to complete the program [16]. Although the patient’s primary cardiologist may be the most important determinant of participation, strong multifaceted endorsement for CR participation from health care providers (e.g., physicians, nurses, exercise physiologists, physical therapists, and social workers) is an important catalyst [14]. Beena and Jimmy (2011) showed that successful HCP–patient communication has a significant impact on patient adherence [17]. Consequently, Well-Beat’s recommendation engine focused on empowering the HCP-patient interaction and used the Toolbar to improve communication.

Based on the theory of planned behavior (TPB), motivational letters sent to post-myocardial infarction patients aimed at influencing their attitude towards attending the CR create subjective norms, and perceived behavioral control. The results showed that attendance rates for the intervention group were substantially higher than those of the control group [18]. In addition, Ouellette and Wood (1998) demonstrated that when a person repeats a behavior often enough, the behavior becomes habitual rendering their intentions less consequential [19]. Based on this insight, Well-Beat’s engine generated personalized text messages that were sent to each patient encouraging them to persevere with the CR program–that is, emphasis was placed on encouraging behavior as opposed to attempting to engage in attitude change.

Armon and Toker (2012) suggested that personality traits are related to both health outcomes including those associated with cardiovascular disease and the likelihood of adopting preventive behaviors [20]. Therefore, personality traits should also be considered in the design and implementation of health-promoting interventions. Moreover, creating a match between the message and the patient’s values makes the patient more committed and increases the effectiveness of the message [21]. Similarly, the New England Institute of Health (2009) suggested that patient heterogeneity should be considered: in addition to differences in knowledge and understanding of the treatment instructions, patient heterogeneity in needs, lifestyle, beliefs, and the tendency to persevere should be addressed [22]. To increase adherence, any intervention should be tailored to the patient’s preferences and behavioral profile.

In addition, the literature introduces other methods of Regime Management [23, 24]; and Incentives [25, 26]. In most cases, though, regime management and incentives alone are not sufficient to generate long-term habits.

The results of the current study provide strong evidence for the impact of the patient-tailored intervention approach on increasing patient adherence to the CR program. The Well-Beat engine and Toolbar employ Cognitive-Behavioral interventions based on the TPB theory and Regime Management. Although all Well-Beat’s interventions are communication-driven, they differ in tone, narrative, and content both between patients and dynamically over time within a patient. The adjustment of the intervention for each patient is based on a multidimensional diagnosis of their profile (e.g., which consists of personality, coping style, readiness to change, and more), along with their behavior. Based on a real-time Machine Learning (ML) algorithm, the intervention recommendation changes automatically and is immediately tailored to the most current information about a patient’s behavior or any change in their profile. For example, a patient exhibiting development in self-efficacy as a result of a successful experience will lead to a change in recommended communication.

Most previous studies focused on improving patient adherence through human-enacted interventions. The current study demonstrates the impact of technology-driven interventions on patient adherence (both indirect—guidance to HCPs, and direct—personalized text messages). In fact, the findings raise interesting questions about the necessity of a face-to-face meeting for some of the patients. This is underscored by the findings that only 51% of patients perceived the in-person sessions as necessary for them and about 40% consider in-person appointments desirable but not necessary. If remote interventions are sufficient in motivating some patients to action and these can be identified in advance, then a whole new treatment regime might be possible. The current findings suggest that using a supportive technology system can enable remote interventions, pointing to a potential breakthrough in service provision to at-risk patients who must avoid healthcare centers, for example, during incidents such as the Covid-19 pandemic. Moreover, using a supportive technology system can substantially reduce the annual costs and burdens of healthcare provision.

The Well-Beat platform can identify patients’ cognitions regarding their experience of a stressful situation (medical condition). It does so based on screening specifically aimed at identifying mental schemas, how a person views their situation, what motivates them, and what impedes them on their way to improvement. The Well-Beat platform sends motivational content to patients to keep them engaged and to motivate them toward adopting more desirable behaviors. This is based, in part, on the notion that a certain level of stress-induced arousal is beneficial for a person when the goal is to improve their performance (in the case of most patients—adherence to treatment, nutrition, and so on). To identify emotional and cognitive patterns and tailor content to patient profiles, the platform must first receive information regarding coping strategies. This information is acquired through questionnaire analysis. It allows the platform to determine, based on the stress appraisal model [10], whether a person is engaging in problem or emotion-focused coping and accordingly determine which type of communication will work better for them. These coping paths help the platform make determinations regarding the emotional and cognitive patterns of patients, and to personalize content accordingly. This information, together with personality-related screening questions, also contributes to general profiling pertaining to different types of personas and the motivators and barriers patient may have when faced with a stressful experience. The platform focuses on motivating patients toward adopting desirable behaviors. Motivational intervention is done whether the patients respond with the desired action, an unwanted action, or refrain from the action at all.

### Clinical implications

The Well-Beat platform demonstrates a novel application of the theoretical principles set forth by Lazarus and Folkman’s (1984) transactional model of stress and coping [10]. By applying these principles, the platform can identify cognitions, coping styles, and attitudes, and based on this produce and recommend personalized motivational content for patients. This paper does not aim to develop new theories. Rather, the results contribute to the body of knowledge by testing the applicability of existing theories to the important and much needed area of patient adherence. The experiment described employs novel analytic methods to enable the application of the transactional model of stress and coping combined with the standard personality and motivational classifications.

While the literature presents various methods for behavior change and increased adherence that can be used to increase CR presence, the results obtained in this study suggest that using personalized interventions can improve adherence with no change to the therapeutic regime. Consequently, instead of seeking the overall "best intervention", one should seek the most effective intervention for *each* patient at a particular point in time. An intervention that is very effective for one patient might be ineffective, or worse, for another, and even the same intervention at the beginning of treatment may fall short later as the patient evolves. Moreover, this study demonstrated that behavioral change can be achieved by reinforcing desired behavior (CR presence activity) and encouraging action in cases where desired behavior does not exist.

The system was developed in the context of medication adherence and this study was conducted to examine whether it can also be useful to motivate patients in the context of exercise. The model used in the current study may possibly be generalized to other chronic or acute conditions where patient behavior over time plays a significant role in preventing the aggravation of their clinical condition. Increasing adherence through personalized care can greatly improve outcomes for all parties involved.

These encouraging results suggest that using personalized interaction aids including both personalized caregiver-patient dialog assistance and personalized text messages can significantly improve patient adherence to the CR program. Furthermore, presenting insights about each patient to the HCP and generating personalized communication along with motivating them to action through text messages can be instrumental for remote care.

### Limitations

Because of data limitations, socioeconomic variables were not included in the current study. Thus, although recruitment was done on a random basis from cardiovascular patients that were offered a free-of-charge three-month rehabilitation program, the effect of socioeconomic status on the results cannot be assessed. The effect of clinical data such as clinical severity, risk factors, and other comorbidities was not examined in this study, as the primary objective was to indicate a comprehensive effect of personalized messages on all patients. However, it can be expected that these factors may influence patient behavior, and in the future, should be addressed in the definition of personal interventions. Additionally, it is possible that the method of recruiting participants influenced its results by creating an unconscious pre-selection of early tendency to adhere. To examine this issue, more research is needed. Finally, the un-blinded nature of this study with HCPs may lead to bias. It is advisable to conduct further studies to confirm the ability to identify the right communication intervention for each patient and to prove the effectiveness of personalized interaction intervention.

### Future studies

This study contributes to the new and evolving field of artificial intelligence interface in healthcare and raises the question of whether an approach based on personalized communication can replace other interventions. To answer this question, future research should focus on cost-effectiveness type comparative studies. The significant differences in outcomes invite further studies that will investigate the impact of personalized interventions on other audiences and additional contexts.

In 2020, two other pilots were carried out in the USA. Unfortunately, those were cut short due to the pandemic and therefore did not lead to publishable results. However, currently, the system is being implemented in an Israeli HMO and is being used to increase nutritional persistence among patients with metabolic diseases. An article describing the results of this study is expected to be published in 2023. Despite the results that showed intensity improvement in terms of adherence, the system was not yet assimilated into the cardiac rehabilitation center due to budgetary considerations in general and the outbreak of the COVID-19 epidemic.

## Appendix A

### Patients’ first engagement

**Fig 3:**
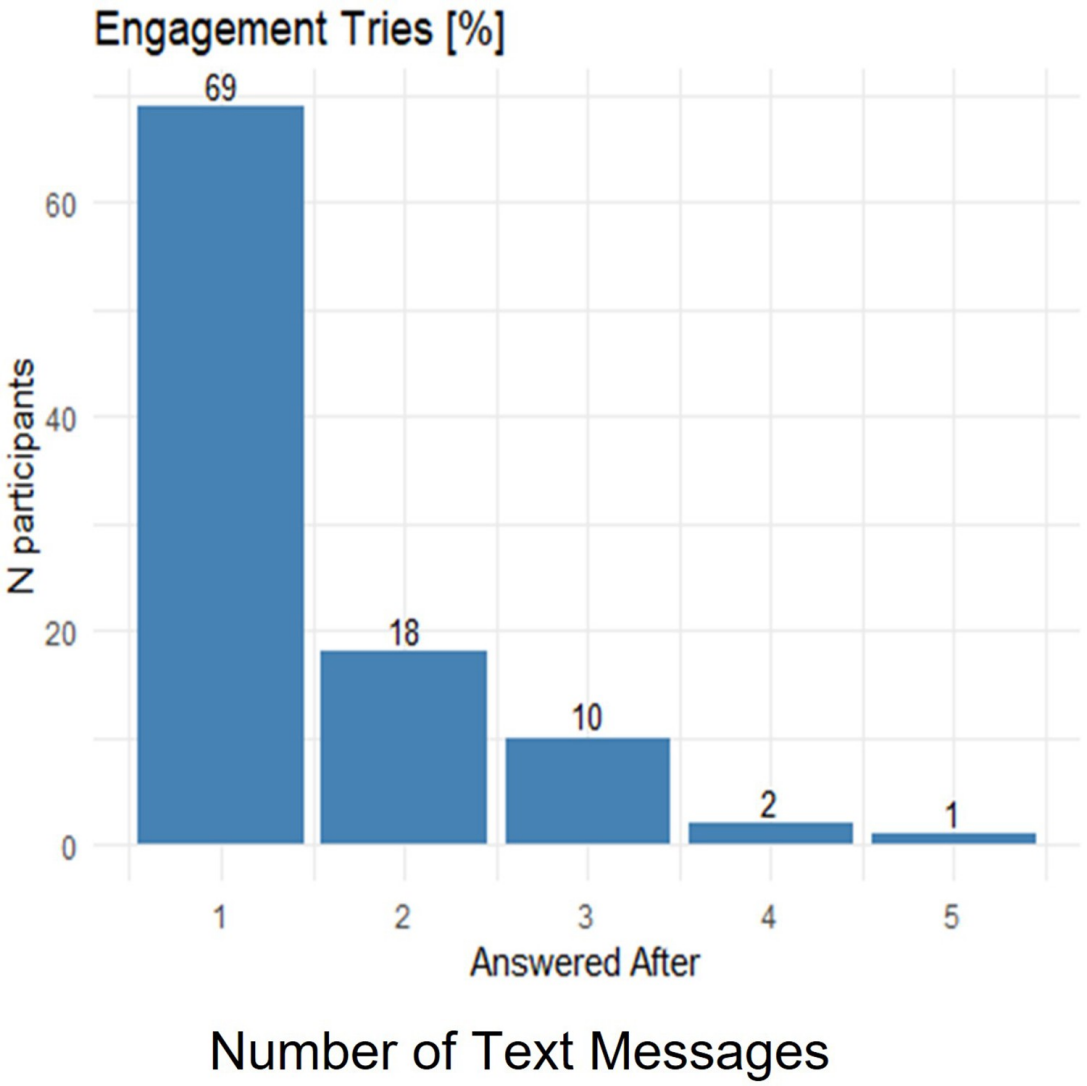
Engagement (answer the Qu.).

Overall, 94% of patients who signed a consent form to participate in the study, completed the questionnaire. As detailed in Fig 3, 69% of participants did so in response to the first text message (74 patients), and another 18% responded to the second text message (20 patients). The high adherence to the questionnaire might indicate a need to be seen, as defined by the patients: *"finally there is a medical system that wants to see me and get to know me and not just the disease" (E*.*G*, *male*, *age 58)*. Another need expressed by patients is assistance in adherence and persistence: *"I wish you could help me persist" (A*.*M*, *male*, *age 71)*. *"I know myself and I know that it will be hard for me to persist*, *I hope you will help me find the strength to do so" (J*.*O*, *female*, *age 45)*. Those needs were expressed repeatedly by most patients regardless of their gender, age, and cardiac history.

## Appendix B

### Patient behavioral patterns

Ongoing patient monitoring makes it possible to identify several behavior patterns. One behavioral pattern was described by Well-Beat as "Need a Jump Start". This pattern is described as a lack of consistency in participation in the first two to three weeks of the process and then participation continuously and regularly. Another pattern of behavior was termed "Stunned by reality" and depicts participation in the CR program in the first four to five weeks and then occasional absences. As a result of the early detection of patterns and the ability to predict them, the CR center will be able to utilize its resources wisely.

## Appendix C

### HCP perceptions

**Fig 4:**
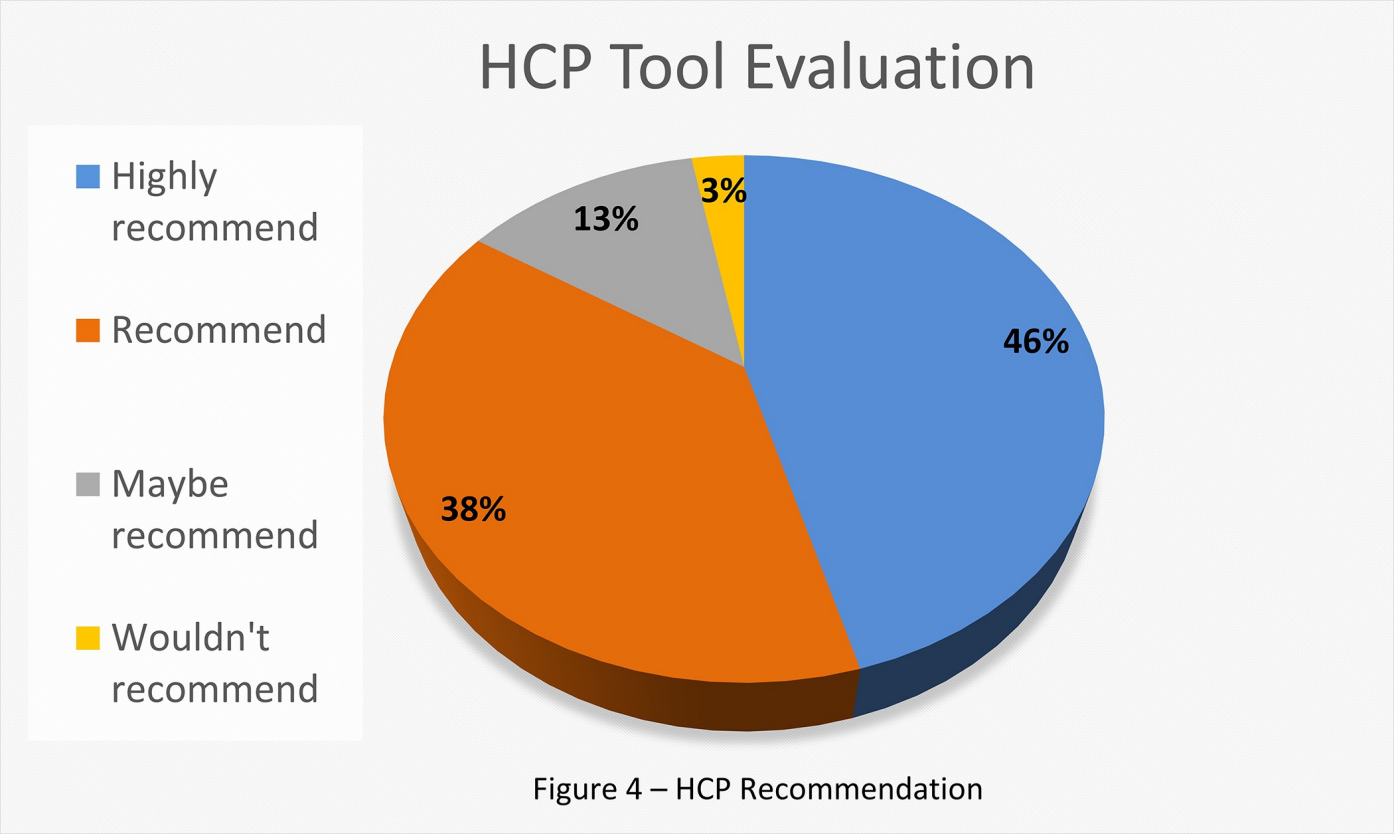
HCP recommendation.

The percentage of recommendations to implement the tool for daily use was extremely high (see Fig 4). After the study ended, the HCPs added that on occasion the system presented them with opportunities for discourse they had not previously thought of. This discourse allowed them to relate to barriers and fears that patients do not always admit to. "We are using experience and intuition, but a tool that gives me a recommendation that takes into account all the relevant characteristics that are in the patient’s subconscious is strengthening my confidence in decisions and causes more attention to specific issues" (A. N., Registered Nurse, CR, Sheba Hospital). "Some patients can benefit and be satisfied with family involvement while others are angry at me for just raising this possibility. The same is true for Group therapy. Therefore, it is very helpful to understand what the patient’s sensitivities are, without using trial and error" (S. I., PT, CR, Sheba Hospital).

After each intervention, HCPs who had a face-to-face dialog with a patient provided feedback about the accuracy of the recommendation presented in the Toolbar. Within 14 weeks of activity, 440 feedback entries were received from the therapists. The HCPs perceived the approach, self-efficacy, maturity, and main driver barrier to be accurate over 90% of the time (93–99% for various parameters) and the barrier parameter accuracy to be around 86%.

Alongside the patient profile, several personalized motivational statements were displayed for use during the caregiver-patient dialog, adjusting the approach, narrative, and content of communication to both the patient’s profile and his/her behavior. Regardless of their prior training, caregivers can use these guidelines to create a conversation that is tailored to each patient and motivates them to action. This was perceived by the center’s management as an additional benefit in implementing the recommendation system as a standard of care. "A tool that presents different layers of information about the patient creates a policy of addressing emotional needs and a uniformity of discourse. Exposure of the team (doctors, nurses, medical physiologists, fitness trainers, dietitians, and medical psychologists) to these creates additional awareness that certainly has a good effect on the process" (Prof. Robert Klempfner, Cardiac Rehabilitation & Prevention Institute director and Sheba Innovation Center scientific director).

## Supporting information

S1 File Test-control data. Supporting data for Table 1.
(CSV)

S2 File Supporting data for Fig 2. (XLSX)

S3 File Supporting data for Fig 3. (XLSX)

S4 File Supporting data for Fig 4. (XLSX)
